# Development of a learning quality scale and associated factors among international medical students in China

**DOI:** 10.1016/j.jtumed.2026.09.004

**Published:** 2026-09-30

**Authors:** Lei Zhong, Jiyang Zhao, Ming Fang, Lei Liu, Jiaqi Mu, Jinzhong Liu, Tong Zheng, Yue Gao, Hao Sun

**Affiliations:** a The Sixth Affiliated Hospital of Harbin Medical University, Harbin, Heilongjiang, China; b Harbin Medical University, Harbin, Heilongjiang, China; c Qiqihar Medical University, Qiqihar, Heilongjiang, China; d The Third Affiliated Hospital of Qiqihar Medical University, Qiqihar, Heilongjiang, China

**Keywords:** العوامل المرتبطة, طلاب الطب الدوليين, جودة التعلم؛ التعليم الطبي, القياس النفسي, تطوير المقاييس, Associated factors, International medical students, Learning quality, Medical education, Psychometrics, Scale development

## Abstract

**Objectives:**

The aims of this study were to develop and validate the International Medical Students’ Learning Quality Scale (IMS-LQS) in China, and to examine student-related factors associated with learning quality.

**Methods:**

A cross-sectional study was conducted among international students enrolled in clinical medicine programs in China. A multicenter convenience sampling strategy was applied at 16 medical universities located in selected regions of China. A preliminary IMS-LQS was developed through literature review, expert consultation, and student interviews. In total, 472 valid questionnaires were collected and randomly divided into an exploratory sample (n = 189) and confirmatory sample (n = 283). Exploratory factor analysis was conducted for item reduction and factor structure exploration, and confirmatory factor analysis was applied to examine model fit and construct validity. Internal consistency, convergent validity, and discriminant validity were assessed. Univariate and multivariate linear regression analyses were performed to identify factors associated with learning quality.

**Results:**

Nineteen items were retained in the final IMS-LQS covering five domains: professional knowledge, clinical skills and practice, professional literacy and ethics, Chinese communication and intercultural competence, and academic and self-directed development. The five-factor solution explained 73.37% of the total variance. The overall Cronbach's alpha coefficient was 0.889, and the domain-level coefficients ranged from 0.817 to 0.851. Confirmatory factor analysis showed that the model fit was acceptable (χ^2^/df = 2.26, comparative fit index = 0.927, Tucker–Lewis index = 0.913, root mean square error of approximation = 0.067, and standardized root mean square residual = 0.056), with adequate composite reliability, average variance extracted, and discriminant validity. Multivariate analysis showed that native English language, longer duration of study in China, motivation related to the cultural environment, and clinical internship experience were factors associated with higher learning quality scores.

**Conclusions:**

The 19-item IMS-LQS had a clear five-domain structure and acceptable reliability and validity among international medical students in China. This scale may support learning quality assessment and targeted educational support in international medical education.

## الملخص

**أهداف البحث:**

هدفت هذه الدراسة إلى تطوير والتحقق من صحة مقياس جودة التعلم لطلاب الطب الدوليين (IMS-LQS) في الصين، وفحص العوامل المتعلقة بالطلاب والمرتبطة بجودة التعلم.

**طرق البحث:**

أُجريت دراسة مقطعية بين الطلاب الدوليين المسجلين في برامج الطب الكلي في الصين. واستُخدمت استراتيجية أخذ عينات عشوائية متعددة المراكز عبر 16 جامعة طبية. جرى تطوير مقياس أولي من خلال مراجعة الأدبيات، واستشارة الخبراء، ومقابلات الطلاب. جُمع ما مجموعُه 472 استبياناً صحيحاً، وقُسِّمت عشوائياً إلى عينة استكشافية (*n* = 189) وعينة توجيهية تأكيدية (*n* = 283). استُخدم تحليل العوامل الاستكشافية لتقليص البنود واستكشاف بنية العوامل، بينما استُخدم تحليل العوامل التوكيدية لفحص مطابقة النموذج والصدق البنائي. كما جرى تقييم الاتساق الداخلي، والصدق التقاربي، والصدق التمايزي، وأُجريت تحليلات الانحدار الخطي أحادي ومتعدد المتغيرات لتحديد العوامل المرتبطة بجودة التعلم.

**النتائج:**

احتفظ المقياس النهائي بـ 19 بنداً وتضمن خمسة مجالات: المعرفة المهنية، والمهارات والممارسات السريرية، والوعي المهني والأخلاقيات، والتواصل الصيني والكفاءة بين الثقافات، والتطور الأكاديمي والذاتي. فسر حل العوامل الخمسة %73.37 من التباين الإجمالي. بلغ معامل ألفا كرونباخ الإجمالي 0.889، وتراوحت المعاملات على مستوى المجالات بين 0.817 و0.851. أظهر تحليل العوامل التوكيدية مطابقة مقبولة للنموذج ($\chi^{2}/df=2.26$، CFI=0.927، TLI=0.913، RMSEA=0.067، و SRMR=0.056)، مع موثوقية مركبة كافية، ومتوسط تباين مستخرج، وصدق تمايزي. أظهر التحليل المتعدد المتغيرات أن اللغة الإنجليزية كلغة أم، وطول فترة الدراسة في الصين، والدافع المرتبط بالبيئة الثقافية، وتجربة التدريب السريري كانت مرتبطة بدرجات أعلى في جودة التعلم.

**الاستنتاج:**

أظهر مقياس جودة التعلم المكون من 19 بنداً بنية واضحة من خمسة مجالات وموثوقية وصدقاً مقبولين بين طلاب الطب الدوليين في الصين، مما قد يدعم تقييم جودة التعلم والدعم التعليمي المستهدف في التعليم الطبي الدولي.

## Introduction

The internationalization of medical education has increased the number of international students enrolled in medical programs in China.^1^ Many international medical students in China are enrolled in English-medium Bachelor of Medicine and Bachelor of Surgery programs, and students in these programs may experience additional communication and professionalism challenges during their clinical training.^2^ These students contribute to academic exchange and the global development of medical education, but they also face distinct learning challenges in a non-native language and cross-cultural educational environment.^3^ Compared with domestic students, international medical students may experience greater difficulties in classroom communication, understanding teaching approaches, integrating theoretical knowledge, participating in clinical learning, and adapting to local academic expectations.^4^ These challenges may affect their learning experience, academic performance, and preparation for clinical training.^5^

Learning quality is an important indicator of the learning status and educational development of students. In medical education, learning quality is not limited to knowledge acquisition, but also includes the development of clinical reasoning, professional attitudes, ethical awareness, communication ability, cultural adaptation, and self-directed learning.^6^ These dimensions are consistent with established descriptions of professional competence in medical education.^7^^,^^8^ The learning quality of international medical students in China may be influenced by their language proficiency, cultural adaptation, learning motivation, previous educational background, duration of study in China, and exposure to clinical practice.^9^ Therefore, a structured and context-specific assessment tool is needed to evaluate the perceived quality of learning in this population.

The existing instruments used to assess student learning have mainly been developed in Western educational settings or designed for domestic students. Thus, these tools may not fully reflect the cross-cultural and language-related demands of medical education in China.^10^, ^11^, ^12^ In addition, few studies have focused on the combined assessment of professional knowledge, clinical skills, professional literacy and ethics, Chinese communication and intercultural competence, and academic and self-directed development among international medical students in China.

Therefore, the aims of this study were to develop and validate the International Medical Students’ Learning Quality Scale (IMS-LQS) in China, and to examine student-related factors associated with perceived learning quality. The IMS-LQS may support the assessment of perceived learning quality and the provision of targeted academic support for international medical students in clinical medical education programs.

## Materials and Methods

### Study design and participants

A cross-sectional design was employed for scale development, psychometric validation, and associated factor analysis. A multicenter convenience sampling strategy was used. The survey link was distributed online through the participating universities, and the final sample included international medical students from 16 medical universities in China. The anonymous structured questionnaire was administered through Wenjuanxing (https://www.wjx.cn/). Data were collected between June 29 and December 30, 2025. In total, 489 submitted questionnaires were screened but 17 were excluded due to incomplete responses or obvious logical inconsistencies, thereby leaving 472 valid questionnaires for the final analysis, corresponding to a valid questionnaire rate of 96.5%.

The total sample was randomly divided into two independent subsamples to examine the factor structure and validate the scale. The exploratory sample included 189 participants and was used for item reduction and exploratory factor analysis. The confirmatory sample included 283 participants and was applied for confirmatory factor analysis and validity testing. The preliminary IMS-LQS contained 25 items and the exploratory sample of 189 participants provided approximately 7.6 participants per item, exceeding the commonly cited minimum ratio of five participants per item required for exploratory factor analysis.^13^ These sample sizes were considered acceptable for the planned exploratory and confirmatory analyses. This study was approved by the institutional ethics committee (approval number: LC2024-052) and was conducted in accordance with the Declaration of Helsinki. Participation was voluntary and anonymous, and informed consent was obtained from all participants before completing the questionnaire.

### Scale development

The initial IMS-LQS was developed through literature review, expert consultation, and student interviews. The IMS-LQS was designed as a student-reported measure of perceived learning quality. Five theoretical domains were initially considered: professional knowledge, clinical skills and practice, professional literacy and ethics, Chinese communication and intercultural competence, and academic and self-directed development. These domains are briefly referred to as Domain 1 to Domain 5 in the tables.

An initial item pool was generated according to these five domains. To improve the relevance of the content, clarity, and cross-cultural applicability, eight experts in medical education, clinical teaching, psychometrics, and language education reviewed the items over two rounds. The research team revised the item wording, conceptual clarity, and domain allocation according to feedback from the experts. In addition, semi-structured interviews were conducted with ten international medical students to assess the comprehensibility and cultural applicability of the items. A 25-item preliminary IMS-LQS was formulated based on this process.

Each item was rated on a five-point Likert scale, ranging from 1 indicating strongly disagree to 5 indicating strongly agree. Higher scores indicated higher perceived learning quality. The total score was calculated by summing the item scores, where higher total scores denoted higher perceived learning quality.

### Data collection and variables

In addition to the preliminary IMS-LQS, the questionnaire collected background characteristics of students that may be associated with perceived learning quality. These variables included sex, age, region of origin, whether English was the native language, academic stage, duration of study in China, previous international study or work experience, source of tuition support, motivation for studying medicine in China, clinical internship experience, and willingness to participate in clinical internship.

### Statistical analysis

Data were analyzed using R software version 4.5.1 and SPSS software version 26.0. Categorical variables were expressed as frequencies and percentages, and continuous variables as the mean and standard deviation. Differences between the exploratory and confirmatory samples were examined using the independent-samples *t*-test, chi-square test, or Fisher's exact test, as appropriate.

Internal consistency was assessed using Cronbach's alpha coefficient and corrected item–total correlation. Suitability of the data for factor analysis was evaluated using the Kaiser–Meyer–Olkin test and Bartlett's test of sphericity. Exploratory factor analysis was performed to explore the factor structure. The number of factors was determined based on eigenvalues and theoretical interpretability. Items with insufficient primary loadings or substantial cross-loadings were removed. Confirmatory factor analysis was then conducted to examine the factor structure. Model fit was evaluated using the chi-square statistic divided by the degrees of freedom, comparative fit index, Tucker–Lewis index, root mean square error of approximation, and standardized root mean square residual. Convergent and discriminant validity were assessed using the standardized factor loadings, composite reliability, average variance extracted, and Fornell–Larcker criterion.

To identify factors associated with perceived learning quality, univariate and multivariate linear regression analyses were performed using the total IMS-LQS score as the dependent variable. Variables with *p* < 0.05 in the univariate analyses were entered into the multivariate linear regression model. For the regression analyses, duration of study in China was dichotomized as > 2 years versus ≤2 years, and motivation for studying medicine in China was dichotomized as cultural environment attraction versus other motivations. All tests were two-sided, and *p*-values less than 0.05 were considered to indicate statistically significant differences.

## Results

### Characteristics of participants

In total, 472 international medical students were included, with 189 participants in the exploratory sample and 283 in the confirmatory sample. The mean age was 26.7 ± 5.7 years, and 246 participants (52.1%) were male. Most participants were from Asia (57.8%), and 222 (47.0%) were undergraduate students. In addition, 226 participants (47.9%) had studied in China for more than two years, 238 (50.4%) had clinical internship experience, and 312 (66.1%) were supported by scholarships. No significant differences were observed between the exploratory and confirmatory samples for any baseline characteristic (all *p* > 0.05), indicating good comparability between the two subsamples (Table 1).

**Table 1:** Characteristics of study participants.

Characteristic	Total sample (n = 472)	Exploratory sample (n = 189)	Confirmatory sample (n = 283)	*p*
Sex				0.347
Male	246 (52.1)	93 (49.2)	153 (54.1)	
Female	226 (47.9)	96 (50.8)	130 (45.9)	
Age, years	26.7 ± 5.7	26.1 ± 5.4	27.0 ± 5.8	0.089
Region of origin				0.375
Asia	273 (57.8)	103 (54.5)	170 (60.1)	
Africa	105 (22.2)	48 (25.4)	57 (20.1)	
Europe	39 (8.3)	16 (8.5)	23 (8.1)	
Americas	51 (10.8)	19 (10.1)	32 (11.3)	
Oceania	4 (0.8)	3 (1.6)	1 (0.4)	
Native English speaker				0.287
Yes	121 (25.6)	43 (22.8)	78 (27.6)	
No	351 (74.4)	146 (77.2)	205 (72.4)	
Academic stage				0.588
Undergraduate student	222 (47.0)	84 (44.4)	138 (48.8)	
Master's student	141 (29.9)	61 (32.3)	80 (28.3)	
Doctoral student	109 (23.1)	44 (23.3)	65 (23.0)	
Duration of study in China				0.329
≤1 year	102 (21.6)	35 (18.5)	67 (23.7)	
1–2 years	144 (30.5)	63 (33.3)	81 (28.6)	
>2 years	226 (47.9)	91 (48.1)	135 (47.7)	
Previous international study or work experience				0.232
Yes	293 (62.1)	124 (65.6)	169 (59.7)	
No	179 (37.9)	65 (34.4)	114 (40.3)	
Motivation for studying medicine in China				0.429
Personal development	78 (16.5)	27 (14.3)	51 (18.0)	
External influence	140 (29.7)	61 (32.3)	79 (27.9)	
Cultural environment attraction	254 (53.8)	101 (53.4)	153 (54.1)	
Clinical internship experience				0.244
Yes	238 (50.4)	102 (54.0)	136 (48.1)	
No	234 (49.6)	87 (46.0)	147 (51.9)	
Willingness to participate in clinical internship				0.899
Yes	449 (95.1)	179 (94.7)	270 (95.4)	
No	23 (4.9)	10 (5.3)	13 (4.6)	
Source of tuition support				0.063
Scholarship	312 (66.1)	133 (70.4)	179 (63.3)	
Self-funded	160 (33.9)	56 (29.6)	104 (36.7)	

### Item reduction and exploratory factor structure

The internal consistency of the preliminary 25-item IMS-LQS was acceptable in the exploratory sample, with an overall Cronbach's alpha coefficient of 0.861. The Cronbach's alpha coefficients ranged from 0.812 to 0.854 for the five initial domains, and all corrected item–total correlations were higher than 0.30. These results indicate that the internal consistency of the preliminary items was acceptable for subsequent factor analysis.

The data were suitable for exploratory factor analysis. The Kaiser–Meyer–Olkin value was 0.811, and Bartlett's test of sphericity was statistically significant (chi-square = 1289.62, degrees of freedom = 300, *p* < 0.001). Based on the eigenvalues and theoretical interpretability, a five-factor structure was supported. Six items (Q5, Q9, Q10, Q14, Q24, and Q25) were removed because of cross-loadings or insufficient primary factor loadings.

After item reduction, 19 items were retained in the final IMS-LQS and five factors: professional knowledge, clinical skills and practice, professional literacy and ethics, Chinese communication and intercultural competence, and academic and self-directed development. The primary factor loadings were acceptable for the retained items and the clear factor structure was consistent with the theoretical framework (Table 2). The five-factor solution explained 73.37% of the total variance. The internal consistency was good for the final 19-item IMS-LQS, with an overall Cronbach's alpha coefficient of 0.889. The Cronbach's alpha coefficients ranged from 0.817 to 0.851 for the five domains, and the corrected item–total correlation ranges were acceptable (Table 3).

**Table 2:** Final items and exploratory factor loadings for the learning quality scale.

Domain	Item	Item content	Loading
Domain 1	Q1	I can understand the core theoretical knowledge in medical courses.	0.74
Domain 1	Q2	I can connect related knowledge across different medical courses.	0.82
Domain 1	Q3	I can accurately grasp the key and difficult points in medical courses.	0.71
Domain 1	Q4	I can apply learned knowledge to explain common medical phenomena or problems.	0.68
Domain 2	Q6	I can apply classroom knowledge to case analysis.	0.68
Domain 2	Q7	I can understand common diagnostic and treatment processes in clinical learning.	0.8
Domain 2	Q8	During clinical observation or internship, I can complete learning tasks assigned by teachers.	0.74
Domain 3	Q11	I understand the basic ethical principles that medical students should follow in learning and practice.	0.75
Domain 3	Q12	I can maintain a serious and responsible professional attitude during learning.	0.73
Domain 3	Q13	I understand the importance of respecting patient privacy and rights.	0.81
Domain 3	Q15	In learning and practice, I pay attention to whether my behavior is consistent with my role as a medical student.	0.66
Domain 4	Q16	I can use Chinese to communicate with teachers about basic learning problems.	0.77
Domain 4	Q17	I can understand commonly used medical terms and professional expressions in class.	0.85
Domain 4	Q18	I can communicate with Chinese classmates about learning in an appropriate manner.	0.65
Domain 4	Q19	I can adapt well to the teaching methods used in Chinese medical courses.	0.72
Domain 4	Q20	Even when cultural differences exist, I can readily participate in medical learning.	0.64
Domain 5	Q21	I can actively seek learning resources other than textbooks to help understand course content.	0.69
Domain 5	Q22	I can formulate a clear study plan according to learning tasks.	0.84
Domain 5	Q23	I adjust my learning methods according to my learning situation.	0.79

Note: Domain 1 = professional knowledge; Domain 2 = clinical skills and practice; Domain 3 = professional literacy and ethics; Domain 4 = Chinese communication and intercultural competence; Domain 5 = academic and self-directed development.

**Table 3:** Variance explained and internal consistency of the final IMS-LQS.

Domain	Items	Eigenvalue	Variance (%)	Cumulative variance (%)	Cronbach's alpha	Item–total correlation
Domain 1	4	5.12	26.95	26.95	0.842	0.45–0.68
Domain 2	3	3.21	16.89	43.84	0.817	0.43–0.66
Domain 3	4	2.34	12.32	56.16	0.836	0.44–0.70
Domain 4	5	1.86	9.79	65.95	0.851	0.47–0.72
Domain 5	3	1.41	7.42	73.37	0.823	0.42–0.65
Total scale	19			73.37	0.889	0.41–0.72

Note: Domain 1 = professional knowledge; Domain 2 = clinical skills and practice; Domain 3 = professional literacy and ethics; Domain 4 = Chinese communication and intercultural competence; Domain 5 = academic and self-directed development.

### Confirmatory factor analysis and validity

Confirmatory factor analysis was performed in the confirmatory sample to further examine the five-domain structure of the final 19-item IMS-LQS. The model fit was acceptable, with a chi-square value divided by the degrees of freedom of 2.26, comparative fit index of 0.927, Tucker–Lewis index of 0.913, root mean square error of approximation of 0.067, and standardized root mean square residual of 0.056. The standardized factor loadings ranged from 0.65 to 0.84. The composite reliability ranged from 0.809 to 0.865, and the average variance extracted ranged from 0.563 to 0.603, supporting good convergent validity (Table 4).

**Table 4:** Confirmatory factor analysis and convergent validity of IMS-LQS.

Domain	Item	Standardized loading	Composite reliability	Average variance extracted
Domain 1	Q1	0.74	0.848	0.583
	Q2	0.81		
	Q3	0.75		
	Q4	0.76		
Domain 2	Q6	0.72	0.809	0.586
	Q7	0.69		
	Q8	0.78		
Domain 3	Q11	0.73	0.838	0.564
	Q12	0.8		
	Q13	0.78		
	Q15	0.71		
Domain 4	Q16	0.74	0.865	0.563
	Q17	0.83		
	Q18	0.65		
	Q19	0.71		
	Q20	0.68		
Domain 5	Q21	0.74	0.82	0.603
	Q22	0.84		
	Q23	0.65		

Note: Domain 1 = professional knowledge; Domain 2 = clinical skills and practice; Domain 3 = professional literacy and ethics; Domain 4 = Chinese communication and intercultural competence; Domain 5 = academic and self-directed development.

Discriminant validity was also supported. The square root of the average variance extracted for each domain was higher than its correlations with the other domains, indicating acceptable discriminant validity for the five domains (Table 5).

**Table 5:** Discriminant validity.

Domain	Domain 1	Domain 2	Domain 3	Domain 4	Domain 5
Domain 1	0.764				
Domain 2	0.493	0.766			
Domain 3	0.436	0.465	0.751		
Domain 4	0.433	0.423	0.484	0.75	
Domain 5	0.458	0.479	0.442	0.411	0.777

Note: Domain 1 = professional knowledge; Domain 2 = clinical skills and practice; Domain 3 = professional literacy and ethics; Domain 4 = Chinese communication and intercultural competence; Domain 5 = academic and self-directed development.

Diagonal values represent the square roots of the average variance extracted, whereas off-diagonal values are the correlations between domains.

### Factors associated with learning quality

The total IMS-LQS score was used as the dependent variable to examine factors associated with learning quality. Univariate linear regression analysis showed that native English language, duration of study in China, motivation for studying medicine in China, clinical internship experience, sex, and source of tuition support were significantly associated with the total IMS-LQS score.

The results showed that being a native English speaker, studying in China for more than two years, being attracted by the cultural environment as the motivation for studying medicine in China, and having clinical internship experience remained significantly associated with higher IMS-LQS scores. By contrast, sex and source of tuition support were not independently associated with the IMS-LQS scores according to the multivariate model (Table 6).

**Table 6:** Factors associated with IMS-LQS scores.

Variable	B	Standard error	β	95% CI	*p*
Constant	41.87	2.48	–	37.01 to 46.73	<0.001
Native English speaker: Yes versus no	3.92	1.18	0.30	1.61 to 6.23	0.001
Duration of study in China: >2 years versus ≤2 years	3.05	0.94	0.25	1.21 to 4.89	0.002
Motivation: cultural environment attraction versus others	2.28	0.91	0.18	0.50 to 4.06	0.013
Clinical internship experience: Yes versus no	2.61	1.07	0.22	0.51 to 4.71	0.016
Sex: Male versus female	1.16	0.85	0.09	−0.51 to 2.83	0.176
Source of tuition support: Scholarship versus self-funded	1.03	0.88	0.08	−0.69 to 2.75	0.244

Note: B = unstandardized coefficient; β = standardized coefficient; 95% CI = 95% confidence interval.

## Discussion

In this study, the IMS-LQS was developed and validated for assessing learning quality among international students enrolled in clinical medicine programs in China, and factors associated with learning quality were examined. The final IMS-LQS retained 19 items and covered five domains: professional knowledge, clinical skills and practice, professional literacy and ethics, Chinese communication and intercultural competence, and academic and self-directed development. Exploratory factor analysis and confirmatory factor analysis both supported this five-domain structure. The IMS-LQS and its domains exhibited good internal consistency, convergent validity, and discriminant validity. These findings indicate the acceptable structural stability and measurement reliability of the IMS-LQS, and it may be applied as a preliminary tool for assessing learning quality among international medical students. The procedures used in this study are consistent with commonly recommended approaches for scale development, construct validation, internal structure assessment, and reliability evaluation in health professions education and related health research.^14^, ^15^, ^16^, ^17^, ^18^

Previous studies have described academic, linguistic, clinical, and sociocultural challenges among international medical students in China.^19^^,^^20^ Cross-cultural scale development also requires considering linguistic clarity and contextual applicability.^21^ The domains included in the IMS-LQS reflect areas previously addressed in studies of professionalism, cultural competence, communication, and self-directed learning.^22^, ^23^, ^24^, ^25^

Several student-related factors associated with perceived learning quality were also identified in the present study. Multivariate linear regression analysis showed that being a native English speaker, longer duration of study in China, motivation for studying medicine in China, and clinical internship experience were associated with higher IMS-LQS scores. Native English speakers may experience fewer language-related difficulties during English-medium instruction. This interpretation is consistent with previous demonstrations that language barriers can affect clinical learning, medical history taking, and communication with teachers, peers, and patients among international medical students in China.^26^

A longer duration of study in China may be associated with gradual adaptation to the language environment, local culture, and teaching approaches. Intercultural communication training and cultural competency education have also been emphasized as important components of healthcare education, particularly in culturally and linguistically diverse learning and clinical environments.^27^^,^^28^ Clinical internship experience may enhance contextualized learning, professional identity, and practical engagement, thereby improving the understanding of medical knowledge and clinical tasks among students. This interpretation is supported by experiential and workplace learning theories, which emphasize how meaningful participation in authentic clinical environments can connect knowledge acquisition with professional practice.^29^^,^^30^ These findings suggest that perceived learning quality is related to the individual background characteristics of students but may also be influenced by language support, cultural adaptation, and opportunities for clinical practice.

From a practical perspective, the IMS-LQS may provide a useful reference for monitoring international medical education and guiding targeted educational support. By examining scores across individual domains, educators and administrators can identify specific areas where international medical students require additional assistance. Existing reviews of clinical learning environment evaluation instruments suggest that structured assessment can help educators to understand the experiences of learners and improve the organization of clinical education.^31^ For students with lower scores in Domain 1 or Domain 2, medical schools could provide structured academic tutoring, case-based learning, and preparatory clinical-skills training before clerkships. Lower scores in Domain 3 may indicate a need for additional education in professionalism, medical ethics, and patient rights. Transitional medical Chinese courses, bilingual simulated-patient exercises, and communication workshops could be implemented before clinical rotations to address lower scores in Domain 4. Peer-mentoring programs between senior and junior international students, individualized learning-strategy guidance, and structured self-directed learning support may be beneficial for students with lower scores in Domain 5.^32^ Mapping educational interventions directly to the five IMS-LQS domains could improve the specificity and practical value of learning support for international medical students.

This study also had several limitations that should be acknowledged. First, the sample size was sufficient for the exploratory and confirmatory factor analyses, but participants were recruited from 16 medical universities in China. Consequently, the institutional and regional representativeness of the sample may be limited, and the stability and generalizability of the IMS-LQS should be further evaluated in larger and more geographically diverse samples. Second, the cross-sectional design captured the perceived learning quality at only one time point, and thus could not reflect changes over time or establish causal relationships, which is an inherent limitation of cross-sectional studies.^33^ Third, the IMS-LQS is a student-reported measure of perceived learning quality, and the present study did not include external objective educational indicators. Consequently, future studies should examine its external validity against academic performance, clinical skills assessments, and teacher or supervisor evaluations. Fourth, some Domain 2 items refer to clinical observation or internship, so the responses from students without previous clinical exposure may reflect their anticipated readiness rather than experience-based self-assessment. Therefore, future validation studies should examine the performance of the IMS-LQS separately among preclinical and clerkship-stage students. Finally, test–retest reliability was not assessed, and thus future studies are needed to examine the temporal stability of the IMS-LQS.

## Conclusions

In this study, a 19-item IMS-LQS with a clear five-domain structure was developed for international medical students in China, with acceptable reliability and validity. Several student-related factors, including native English language, longer study duration in China, cultural motivation, and clinical internship experience, were associated with higher perceived learning quality. The IMS-LQS may support perceived learning quality assessment and targeted educational support in international medical education.

## Informed consent

Participation was voluntary and anonymous. Informed consent was obtained from all participants before questionnaire completion.

## Ethical approval

This study was approved by the institutional ethics committee (approval number: LC2024-052) and conducted in accordance with the Declaration of Helsinki.

## Authors contributions

LZ: Conceptualization, Supervision, Project administration, Writing – review and editing. WZ: Investigation, Data curation, Writing – original draft. MF: Investigation, Data curation, Methodology. LL: Investigation, Resources, Writing – review and editing. JM: Methodology, Validation, Writing – review and editing. JL: Resources, Supervision, Project administration. TZ: Data curation, Formal analysis, Visualization. YG: Investigation, Data curation, Formal analysis. HS: Conceptualization, Methodology, Formal analysis, Writing – original draft, Writing – review and editing. All authors have critically reviewed and approved the final draft and are responsible for the content and similarity index of the manuscript.

## Data availability

The data that support the findings obtained in this study are available from the corresponding author upon reasonable request.

## Source of funding

Not applicable.

## Conflict of interest

The authors declare that they have no known competing financial interests or personal relationships that could have appeared to influence the work reported in this paper.
